# Beta blocker use and breast cancer survival by subtypes: A population-based cohort study

**DOI:** 10.1016/j.breast.2025.104474

**Published:** 2025-04-08

**Authors:** Oliver William Scott, Sandar Tin Tin, Edoardo Botteri

**Affiliations:** a Department of Oncology, School of Medical Sciences, University of Auckland, Auckland, New Zealand; b Department of Epidemiology and Biostatistics, School of Population Health, University of Auckland, Auckland, New Zealand; c Oxford Population Health, University of Oxford, Oxford, UK; d Department of Research, Cancer Registry of Norway, Norwegian Institute of Public Health, Oslo, Norway; e Section for Colorectal Cancer Screening, Cancer Registry of Norway, Norwegian Institute of Public Health, Oslo, Norway

**Keywords:** Breast cancer, Mortality, Beta blockers, Pharmacoepidemiology, Cohort study

## Abstract

**Background:**

The associations between beta blocker (BB) use and breast cancer outcomes have been examined in previous observational studies, however the results are inconsistent. We examine these associations in a large population-based cohort of New Zealand (NZ) women with breast cancer.

**Methods:**

Postmenopausal women diagnosed with a first primary early invasive breast cancer between 2006 and 2020 were identified from the NZ Breast Cancer Foundation National Register and linked to national pharmaceutical data, hospital discharges, and death records. Cox proportional hazard models were used to estimate hazards of breast cancer-specific death (BCD), recurrence free interval (RFI), and distant recurrence free interval (DRFI) associated with BB use at diagnosis. Analyses were stratified by subtype.

**Results:**

Of the 13,535 women included in analyses, 2,238 (17 %) were using a BB at diagnosis and the median follow up time with BCD as the outcome was 5.6 years. BB use (vs non-use) was not associated with BCD (adjusted hazard ratio: 1.03; 0.86–1.23), RFI (HR = 0.94; 0.81–1.09), or DRFI (HR = 0.98; 0.83–1.15) overall. In women with triple negative breast cancer (TNBC), BB use was associated with a significantly longer RFI (HR = 0.71; 0.52–0.98) and DRFI (HR = 0.70; 0.50–0.98), and there was a suggestion of a decreased risk of BCD (HR = 0.74; 0.52–1.06). BB use was also associated with a significantly longer RFI in women with Luminal B HER2+ cancers (HR = 0.52; 0.29–0.92).

**Conclusions:**

Our findings suggest that any protective effect on breast cancer prognosis associated with BB use may be confined to specific subtypes, particularly TNBC.

## Highlights

• Beta blockers may be beneficial for breast cancer, but the evidence is inconsistent.
• Few studies have stratified by tumour characteristics such as subtype.
• Beta blockers may only be beneficial for triple negative breast cancer.
• Beta blockers should continue to be explored as a potential adjuvant therapy for TNBC.

## 1 Introduction

Breast cancer is the most common cancer in women and the leading cause of female cancer mortality worldwide [1]. Comorbidities, particularly cardiovascular disease, are increasingly common in breast cancer patients due to an increasing prevalence of shared risk factors [2]. Examining the association between commonly used cardiovascular medications such as beta blockers (BBs) and breast cancer outcomes is therefore warranted.

Breast cancer survival has improved over the last two decades [3], however the prognosis of some breast cancers (e.g., triple negative breast cancer (TNBC) and late-stage breast cancer) remains poor [4]. Several observational studies examining the association between BB use and breast cancer prognosis have indicated a potential protective effect of BBs, while others have reported a null association or a detrimental effect [5]. The inconsistency of these results may be due, at least in part, to the fact that analyses have not often taken into account important tumour characteristics such as subtype and stage [6]. Therefore, our objective was to explore the relationship between BB use and breast cancer prognosis in a cohort of newly diagnosed breast cancer patients and in patient subgroups defined by subtype and stage.

## 2 Methods

### 2.1 Data sources

**Fig. 1:**
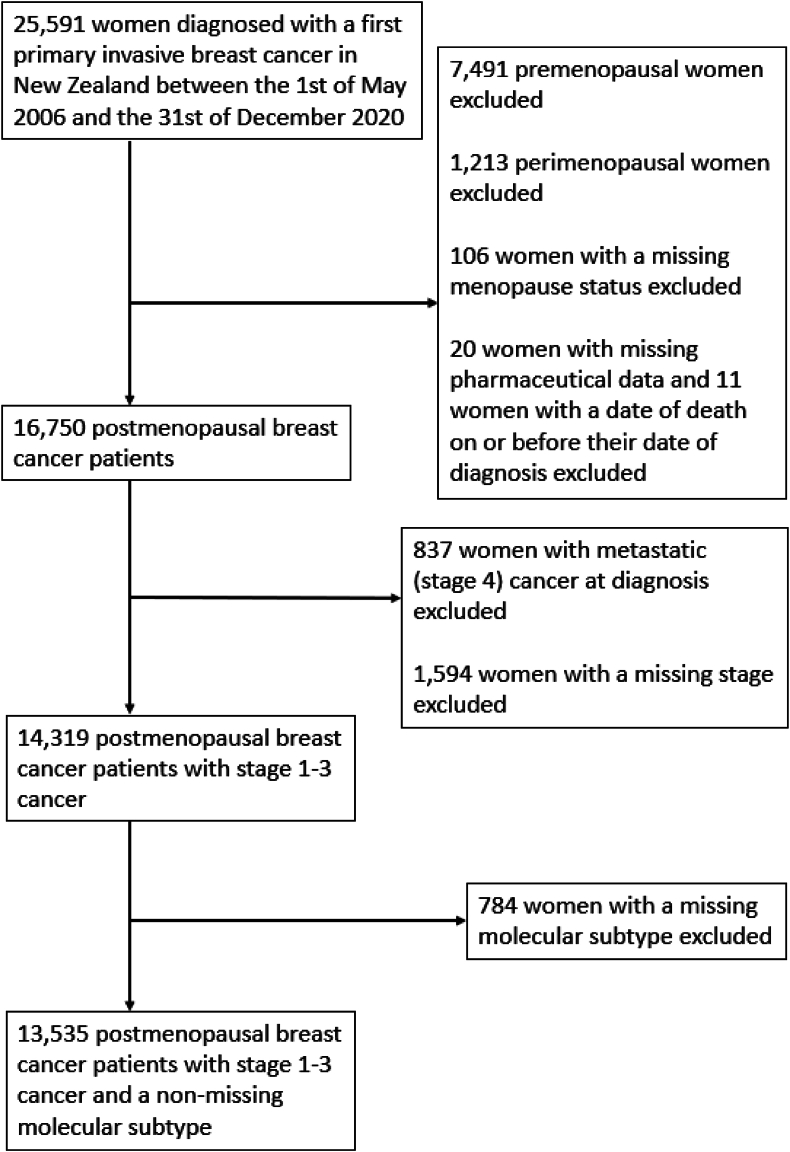
Flow chart of patient selection.

Eligible women were all those with a first primary invasive breast cancer recorded in the New Zealand Breast Cancer Foundation National Register [7] between May 1, 2006 and Dec 31, 2020. This register began in the Auckland region in the year 2000, was extended to the Waikato, Wellington, and Christchurch regions between 2005 and 2010, and was nationalised in 2020. Using an anonymised patient identifier, data were linked to several national data bases: the Pharmaceutical Collection (PHARMS), a national database containing dispensing information from pharmacists for subsidised dispensings [8]; the National Minimum Dataset, relating to all patients discharged from public hospitals [9]; and the National Mortality Collection, with information about all certified deaths [10]. The total number of women eligible for inclusion in our study was 25,591. Because BBs are infrequently used in younger women, we firstly excluded premenopausal and perimenopausal women and women with a missing menopause status to derive a more homogeneous study population. We also excluded women whose records did not link to at least one dispensing from PHARMS or if their date of death was on or before their date of breast cancer diagnosis. We then excluded patients with metastatic disease at diagnosis (as these women have a poor prognosis and are unlikely to benefit from BB therapy), as well as women with a missing stage. Finally, we excluded women with a missing subtype. The final cohort for analyses was comprised of 13,535 women with nonmetastatic breast cancer (Fig. 1).

### 2.2 Exposure and outcome data

In the PHARMS database, medications dispensed in the four-month period prior to diagnosis (including those dispensed on the date of diagnosis) were determined using the therapeutic group ID, a PHARMAC identifier for each group of Anatomical, Therapeutic, and Chemical properties [8]. All BBs dispensed to women in our cohort were included, except those used topically for glaucoma. The four-month period prior to breast cancer diagnosis was chosen because it is likely that women taking BBs during this period were taking them at the time of diagnosis. A diagnosis of cancer as well as subsequent surgery and associated treatments are generally very stressful events for patients [11], and it has been hypothesised that BBs might aid in alleviating this stress [12].

Cause of death was determined from the Breast Cancer Foundation National Register and National Mortality Collection, with ICD codes C50.0 to C50.9 classified as deaths from breast cancer.

### 2.3 Confounders

Demographic and clinical information came from the Breast Cancer Foundation National Register, and covariates considered included date of diagnosis, age, ethnic group, socioeconomic deprivation (New Zealand Deprivation Index, a measure of deprivation based on census data, ranging from 1 (least deprived) to 10 (most deprived)) [13], urban/rural status, public/private status of the surgical treatment facility, region, size of the primary tumour (TNM T category) [14], status of the regional lymph nodes (TNM N category) [14], grade, mode of detection (screen detected vs symptomatic), peritumoral lymphovascular invasion, and subtype (as defined previously [15], including Luminal A (ER+ and/or PR+, HER2-, grade=1), Luminal B HER2- (ER+ and/or PR+, HER2-, grade=2 or 3), Luminal B HER2+ (ER+ and/or PR+, HER2+), HER2+ non-luminal (ER-, PR-, HER2+), and TNBC (ER-, PR-, HER2-)). In New Zealand, ER, PR, and HER2 statuses are determined using immunohistochemistry, and the HER2 FISH test is additionally used to determine HER2 status. Given that the Breast Cancer Foundation National Register does not record the expression of Ki-67, we used tumour grade to distinguish between Luminal A and Luminal B HER2-cancers [16]. We adjusted for the TNM T and N categories separately as they confer more information separately relative to combining them into a composite variable. Other peri-diagnostic medications included statins, non-steroidal anti-inflammatory drugs (NSAIDs), aspirin, angiotensin converting enzyme inhibitors (ACEIs), angiotensin receptor blockers (ARBs), and diuretics. Comorbidities adjusted for included any cardiac condition (angina, arrhythmia, congestive heart failure, hypertension, myocardial infarction, ‘other cardiac conditions’, and valve disease) as yes/no, diabetes, stroke, chronic obstructive pulmonary disorder, and peripheral vascular disease. We defined comorbidities as any of the above conditions appearing in a patient's hospital record in the 5-year period before their breast cancer diagnosis. Year of diagnosis and age were adjusted for as continuous variables, while all other variables were adjusted for as categorical as outlined in Table 1. Cancer treatments such as chemotherapy and radiotherapy were not considered as confounders because these variables are highly correlated with covariates already adjusted for (such as stage and grade).

**Table 1:** Characteristics of breast cancer patients by peri-diagnostic beta blocker use.

Characteristics	Beta blocker use at diagnosis^a^	
	Yes (n (%))	No (n (%))
**Overall**	2,238	11,297
**Year of diagnosis**		
Median (range)	2015 (2006–2020)	2015 (2006–2020)
**Age at diagnosis (years)**		
Median (range)	69 (40–98)	63 (34–96)
**Ethnic group**		
European	1,725 (77)	8,705 (77)
Māori	210 (9)	1,076 (10)
Pacific	119 (5)	575 (5)
Asian	154 (7)	797 (7)
Other	30 (1)	144 (1)
**Deprivation (NZDep)**^b^		
Median (range)	6 (1–10)	5 (1–10)
**Urban/rural status**		
Urban	1,888 (84)	9,592 (85)
Rural	216 (10)	1,240 (11)
Unknown	134 (6)	465 (4)
**Status of surgical treatment facility**		
Public	1,572 (70)	7,085 (63)
Private	521 (23)	3,508 (31)
Unknown	145 (6)	704 (6)
**Region**		
Auckland	1,083 (48)	5,674 (50)
Christchurch	359 (16)	2,050 (18)
Waikato	491 (22)	1,932 (17)
Wellington	305 (14)	1,641 (15)
**Size of the primary tumour (TNM T category)**		
0	3 (0.1)	12 (0.1)
1	1,219 (54)	7,036 (62)
2	859 (38)	3,604 (32)
3	128 (6)	517 (5)
4	27 (1)	113 (1)
Unknown	2 (0.1)	15 (0.1)
**Status of regional lymph nodes (TNM N category)**		
0	1,484 (66)	7,760 (69)
1	110 (5)	625 (6)
2	386 (17)	1,893 (17)
3	153 (7)	617 (5)
4	100 (4)	393 (3)
Unknown	5 (0.2)	9 (0.1)
**Cancer stage (composite)**		
1	1,043 (47)	6,076 (54)
2	884 (40)	3,971 (35)
3	311 (14)	1,250 (11)
**Cancer grade**		
Well differentiated	459 (21)	2,746 (24)
Moderately differentiated	1,129 (50)	5,461 (48)
Poorly differentiated	640 (29)	3,037 (27)
Unknown	10 (0.5)	53 (0.5)
**Method of diagnosis**		
Symptomatic	1,290 (58)	5,290 (47)
Screen detected	948 (42)	6,007 (53)
**Lymphovascular invasion**		
No	1,487 (66)	7,811 (69)
Yes	733 (33)	3,451 (31)
Unknown	18 (1)	35 (0.3)
**Subtype**		
Luminal A	449 (20)	2,664 (24)
Luminal B HER2-	1,274 (57)	6,050 (54)
Luminal B HER2+	178 (8)	979 (9)
HER2+ non-luminal	91 (4)	474 (4)
Triple negative	246 (11)	1,130 (10)
**Other medication use before diagnosis**^a^		
Statins	1,175 (53)	2,345 (21)
NSAIDs	270 (12)	1,482 (13)
Aspirin	923 (41)	1,306 (12)
ACEIs	928 (41)	2,040 (18)
ARBs	369 (16)	737 (7)
Diuretics	700 (31)	1,151 (10)
**Hospitalised comorbidities**^c^		
Any cardiac condition	635 (28)	747 (7)
Diabetes	231 (10)	455 (4)
Stroke	102 (5)	175 (2)
COPD	55 (2)	178 (2)
Peripheral vascular disease	38 (2)	36 (0.3)

Note) The chi square test was statistically significant (p < 0.05) for every variable except for ethnic group and the use of NSAIDs.

a Use of the respective medication in the four-month period prior to breast cancer diagnosis (including those dispensed on the date of diagnosis).

b The NZDep is an area-based measure of socioeconomic deprivation in New Zealand. 1 represents the areas with the least deprived scores and 10 the areas with the most deprived scores.

c Comorbidities included those in a patient's hospital records five years before breast cancer diagnosis. Cardiac conditions included any of angina, arrhythmia, congestive heart failure, hypertension, myocardial infarction, ‘other cardiac conditions’, and valve disease.

### 2.4 Statistical analyses

Comparisons by BB use at baseline (date of diagnosis of breast cancer) were conducted using the chi-square test. We used Cox proportional hazard models to assess hazard ratios (HRs) of breast cancer-specific mortality (BCD) associated with any peri-diagnostic BB use vs non-use. PHARMS coverage was complete to the end of 2021, so we followed patients from their breast cancer diagnosis until death or December 31, 2021.

We also conducted analyses examining breast cancer recurrence (BCR) as the outcome. In the recurrence free interval (RFI) analyses, a BCR was defined as either a local/regional recurrence or distant metastasis, and women were followed from their breast cancer diagnosis until BCR, death from breast cancer, last follow up date, or end of PHARMS coverage, whichever came first. In the distant recurrence free interval (DRFI) analyses, a BCR was defined as a distant metastasis exclusively, and women were followed from their breast cancer diagnosis until distant metastasis, death from breast cancer, last follow up date, or end of PHARMS coverage, whichever came first. In the DRFI analysis, if women had a local/regional recurrence before a distant metastasis, they were followed until their distant metastasis. In both the RFI and DRFI analyses, both BCR and death from breast cancer were counted as event endpoints, while deaths from other causes were censored.

Analyses were firstly conducted on the entire cohort for each outcome, and then stratified by subtype and TNM stage [14]. As the TNBC subgroup was of special interest to us *a priori* [6], we also carried out analyses stratified by stage in women with TNBC.

For the analyses on the entire cohort and in TNBC patients, we carried out sensitivity analyses in which women were required to have had at least two dispensings of a BB in the six-month period prior to diagnosis to be considered a user. In this analysis, women with only one dispensing in the six-month period prior to diagnosis were counted as nonusers. In order to compare BB users to patients using other medications for a similar indication, further sensitivity analyses were carried out comparing BB users to BB nonusers who used another antihypertensive medication (as opposed to comparing BB users to all BB nonusers). We then combined these two analyses in another sensitivity analysis. In a separate analysis, we additionally adjusted for chemotherapy and radiotherapy (both as binary yes/no variables and including both neoadjuvant and adjuvant therapies) to determine if doing so conferred any independent confounding effect. Finally, we carried out sensitivity analyses in pre and perimenopausal women only, in which analyses were again conducted on all women and in women with TNBC.

Results are reported as HRs and their 95 % confidence intervals (CIs), with the two-sided significance level set at 0.05. Statistical analyses were conducted in STATA 17.0 (StataCorp, College Station, TX).

## 3 Results

Median follow up with BCD as the outcome was 5.6 years (range 0.1–15.7 years), with 905 dying of breast cancer, and 1,233 dying from other causes. Of the 13,535 women in our cohort, 2,238 (17 %) were users of BBs at diagnosis (Table 1). BB users compared to nonusers were older, were from more deprived areas, were more likely to have had their surgery in a public facility and be from the Waikato region, had a size of the primary tumour (TNM T category) and stage (composite) of two more often, and were more likely to have had a symptomatic diagnosis**.** BB users were also more likely to have used other medications (statins, aspirin, ACEIs, ARBs, and diuretics) and to have had documented comorbidities (any cardiac condition, diabetes, stroke, and peripheral vascular disease) (p < 0.05 for all differences).

There was no association between the use of BBs and BCD (adjusted hazard ratio: 1.03; 95 % CI 0.86–1.23), RFI (HR = 0.94; 0.81–1.09), or DRFI (HR = 0.98; 0.83–1.15) in the entire cohort (Table 2). When we stratified by subtype, BB use was associated with a statistically significantly longer RFI (HR = 0.71; 0.52–0.98) and DRFI (HR = 0.70; 0.50–0.98) in women with TNBC, and there was a suggestion of a decreased risk of BCD (HR = 0.74; 0.52–1.06) (p values for heterogeneity for TNBC vs all other subtypes combined of 0.04, 0.08, and 0.04 for BCD, RFI, and DRFI respectively). In women with Luminal B HER2+ cancer, BB use was associated with a statistically significantly longer RFI (HR = 0.52; 0.29–0.92). There was no association between BB use and any outcome in women with Luminal A, Luminal B HER2-, or HER2+ non-luminal cancers. When we tested for heterogeneity across all subtypes, there was evidence of effect medication for RFI only (p values for heterogeneity of 0.13, 0.01, and 0.08 for BCD, RFI, and DRFI respectively).

**Table 2:** Associations of breast cancer outcomes with peri-diagnostic use of beta blockers (vs non-use) in breast cancer patients.

**Medication Usage At Diagnosis**	Breast cancer specific death			Recurrence free interval			Distant recurrence free interval		
	**No. Breast cancer deaths**	**No. person-years**	**Adjusted**^a^**HR (95 % CI)**	**No. Breast cancer deaths or recurrences**	**No. person-years**	**Adjusted**^a^**HR (95 % CI)**	**No. Breast cancer deaths or distant metastases**	**No. person-years**	**Adjusted**^a^**HR (95 % CI)**
**Overall association**									
BB nonuser	704	71,051	1.00	1,124	53,186	1.00	928	53,960	1.00
BB user	201	13,312	1.03 (0.86–1.23)	274	10,225	0.94 (0.81–1.09)	242	10,375	0.98 (0.83–1.15)
**By subtype**									
BB nonuser, Luminal A	30	18,353	1.00	101	13,800	1.00	50	13,974	1.00
BB user, Luminal A	10	3,036	1.23 (0.51–2.97)	18	2,353	0.73 (0.41–1.28)	12	2,379	1.00 (0.47–2.09)
BB nonuser, Luminal B HER2-	350	36,781	1.00	561	27,540	1.00	478	27,853	1.00
BB user, Luminal B HER2-	102	7,303	1.14 (0.89–1.47)	147	5,599	1.07 (0.87–1.32)	130	5,672	1.08 (0.87–1.34)
BB nonuser, Luminal B HER2+	61	6,003	1.00	111	4,496	1.00	87	4,591	1.00
BB user, Luminal B HER2+	19	1,035	0.73 (0.35–1.49)	23	802	0.52 (0.29–0.92)	21	812	0.59 (0.31–1.12)
BB nonuser, HER2+ non-luminal	60	3,136	1.00	84	2,292	1.00	76	2,347	1.00
BB user, HER2+ non-luminal	18	497	1.89 (0.84–4.29)	24	371	1.68 (0.87–3.24)	22	385	1.44 (0.70–2.96)
BB nonuser, triple negative	203	6,778	1.00	267	5,057	1.00	237	5,196	1.00
BB user, triple negative	52	1,441	0.74 (0.52–1.06)	62	1,099	0.71 (0.52–0.98)	57	1,127	0.70 (0.50–0.98)
**By stage**									
BB nonuser, stage one	109	39,950	1.00	295	29,850	1.00	165	30,312	1.00
BB user, stage one	21	6,672	0.91 (0.54–1.54)	48	5,107	0.78 (0.56–1.10)	28	5,186	0.85 (0.55–1.33)
BB nonuser, stage two	312	24,015	1.00	456	18,041	1.00	403	18,279	1.00
BB user, stage two	88	4,967	1.09 (0.84–1.42)	115	3,828	1.07 (0.85–1.34)	106	3,875	1.07 (0.84–1.36)
BB nonuser, stage three	283	7,086	1.00	373	5,294	1.00	360	,5370	1.00
BB user, stage three	92	1,673	1.00 (0.76–1.31)	111	1,290	0.90 (0.70–1.15)	108	1,314	0.91 (0.71–1.18)

Note) HR=Hazard Ratio, CI=Confidence Interval.

a Adjusted for year of diagnosis, age, ethnic group, deprivation, urban/rural status, public/private status of the surgical treatment facility, register, size of the primary tumour, status of the regional lymph nodes, grade, mode of detection, lymphovascular invasion, subtype, and other drug use and hospitalised comorbidities (other drugs including statins, NSAIDs and aspirin, ACEIs, ARBs, and diuretics. Comorbidities including any cardiac condition as yes/no, diabetes, stroke, COPD, and peripheral vascular disease). Other drug covariates were modelled in the same fashion as beta blockers (i.e., yes/no in the four months prior to breast cancer diagnosis, including those dispensed on the date of diagnosis).

When we stratified by cancer stage in the entire cohort (Table 2), there was no evidence that stage modified the effect of BB use for any outcome (p values for heterogeneity of 0.79, 0.28, and 0.53 for BCD, RFI, and DRFI respectively). When we stratified by stage in those with TNBC (Table 3), the HRs were lower for every outcome in those with stage three disease, although none of the interaction terms reached statistical significance (p values for heterogeneity of 0.84, 0.29, and 0.66 for BCD, RFI, and DRFI respectively).

**Table 3:** Associations of breast cancer outcomes with peri-diagnostic use of beta blockers (vs non-use) in breast cancer patients, by stage in triple negative women.

**Medication Usage At Diagnosis**	Breast cancer specific death			Recurrence free interval			Distant recurrence free interval		
	**No. Breast cancer deaths**	**No. person-years**	**Adjusted**^a^**HR (95 % CI)**	**No. Breast cancer deaths or recurrences**	**No. person-years**	**Adjusted**^a^**HR (95 % CI)**	**No. Breast cancer deaths or distant metastases**	**No. person-years**	**Adjusted**^a^**HR (95 % CI)**
BB nonuser, stage one	41	3,294	1.00	66	2,414	1.00	50	2,491	1.00
BB user, stage one	7	618	0.90 (0.33–2.42)	10	482	0.75 (0.34–1.66)	7	494	0.73 (0.28–1.91)
BB nonuser, stage two	89	2,667	1.00	112	2,013	1.00	103	2,057	1.00
BB user, stage two	26	622	0.82 (0.49–1.36)	31	460	0.97 (0.61–1.54)	29	477	0.84 (0.52–1.35)
BB nonuser, stage three	73	817	1.00	89	631	1.00	84	648	1.00
BB user, stage three	19	201	0.64 (0.31–1.32)	21	157	0.51 (0.26–1.01)	21	157	0.58 (0.30–1.12)

Note) HR=Hazard Ratio, CI=Confidence Interval.

a Adjusted for year of diagnosis, age, ethnic group, deprivation, urban/rural status, public/private status of the surgical treatment facility, register, size of the primary tumour, status of the regional lymph nodes, grade, mode of detection, lymphovascular invasion, subtype, and other drug use and hospitalised comorbidities (other drugs including statins, NSAIDs and aspirin, ACEIs, ARBs, and diuretics. Comorbidities including any cardiac condition as yes/no, diabetes, stroke, COPD, and peripheral vascular disease). Other drug covariates were modelled in the same fashion as beta blockers (i.e., yes/no in the four months prior to breast cancer diagnosis, including those dispensed on the date of diagnosis).

In the sensitivity analyses in which women were required to have had at least two dispensings of a BB in the six-month period prior to diagnosis to be counted as a user, similar findings were noted (Supplementary Tables 1 and 3). Similar findings were also noted when the comparison group was changed to BB nonusers who used another antihypertensive medication (Supplementary Tables 2 and 3). Similar findings were again noted in exploratory analyses in which we additionally adjusted for chemotherapy and radiotherapy (data not shown). Finally, in the sensitivity analyses in which only pre and perimenopausal women were included, there was no association found between BB use and any outcome in all women or in women with TNBC (Supplementary Table 4).

## 4 Discussion

In this population-based cohort of breast cancer patients in New Zealand, there was no association between peri-diagnostic BB use and BCD, RFI, or DRFI in the entire cohort. However, there was evidence to suggest that BBs were associated with a better prognosis in patients with TNBC. We also showed that BBs may be efficacious in women with Luminal B HER2+ cancer.

Our finding of no association between BB use and breast cancer prognosis in the entire cohort is consistent with the results of a recently published meta-analysis of ours [5]. However, we indicated that BB use was associated with a better prognosis for patients with TNBC in the current study, particularly for the outcomes including recurrence as an endpoint. This finding is consistent with a number of previous studies that have examined patients with TNBC as a subgroup of interest [6,[17], [18], [19]]. The first of these was a 2011 study conducted in the USA, in which the authors reported a longer RFI associated with the use of BBs in TNBC patients (HR = 0.30; 0.10–0.87) [17]. When results from studies that have stratified by subtype were recently meta-analysed, Löfling and others found a HR of 0.74 (0.55–1.00) for the association between BB use and BCD in TNBC patients (in which four studies were pooled), and 0.58 (0.38–0.89) for BCR (in which five studies were pooled) [6].

The biological mechanism relating BB use to improved breast cancer outcomes in patients with TNBC in particular appears to be twofold. In preclinical studies involving mouse models, it has been shown that blocking beta-adrenergic signalling through the use of BBs effectively inhibits the effects of stress and improves breast cancer outcomes, particularly in TNBC [20,21]. Moreover, it has been postulated that stress may modify the efficacy of chemotherapy through beta-adrenergic signalling [22,23]. As such, BB use may interact with chemotherapy to elicit a more protective effect, and because TNBC patients receive chemotherapy more often than patients with other subtypes [24], this potential synergistic effect may manifest more often in patients with TNBC.

There was a protective association shown for Luminal B HER2+ patients for RFI. This result may be partly explained by the fact that Luminal B cancers have a high proliferation index [25], meaning that any potential protective effect of BBs may be easier to observe in this patient subgroup. However, other subtypes which also have a high proliferation index (such as HER2+ non-luminal [26]) did not show results which were concordant with those for Luminal B HER2+ cancers. We cannot rule out the possibility of our results in Luminal B HER2+ cancers being derived by way of chance alone.

The associations of BB use with all cancer outcomes did not differ by stage in this study, as reported previously [6,27]. However, the HRs were lower for those with stage three disease in TNBC patients, which is consistent with a recent observational study that found a protective association in TNBC patients for regional disease, but not for local disease [6]. Late stage breast cancer (and TNBC in particular) is an aggressive form of disease with a high potential for metastasis [4], and given that the potential protective effect of BBs may be exclusive to TNBC [6], the effect may be most apparent in this patient subgroup.

The primary strength of our study is that we had a large cohort of breast cancer patients sourced from a high quality population-based database. The Breast Cancer Foundation National Register has been checked against the National Cancer Registry (which contains information on all cancers, including breast cancer) and has been found to be at least 99 % complete, and the registry data we used contains more comprehensive and accurate information than the national data sources [[28], [29], [30]]. Our pharmaceutical data were derived from a high quality and automated national database, and there was no recall bias [31] associated with medication records as a result. Furthermore, New Zealand records medication dispensings instead of prescriptions [8], which are a stronger proxy for medication adherence. Finally, we avoided the introduction of immortal time bias [32] by exclusively classifying BB use in the pre-diagnostic period.

Our study also has limitations. We did not have access to primary care data, which meant that our comorbidity data were restricted to hospital admissions in the relevant timeframe. Furthermore, this limited access to a range of other potential confounders. The most commonly prescribed BB in New Zealand is a selective BB, metoprolol [33], and we therefore did not have the power to explore the relationship between non-selective BBs and breast cancer outcomes. Several preclinical studies have suggested that non-selective BBs may have a higher efficacy in inhibiting pathways involved in breast cancer progression and metastasis [21,34]. It is worth noting that some of our analyses (such as examining the effect of BBs in TNBC by stage) were limited by relatively small numbers and thus low statistical power. Finally, although TNBC is a subtype of breast cancer that occurs more frequently in premenopausal women [35], we had limited power to explore the effect of BBs in this patient group due to their low rate of BB use.

In conclusion, we found that any protective effect on breast cancer prognosis associated with BB use may be confined to specific subtypes, particularly TNBC. As treatment options for patients with TNBC remain expensive and/or unavailable, BBs should continue to be explored as a potential avenue of treatment for this patient population.

## CRediT authorship contribution statement

**Oliver William Scott:** Writing – review & editing, Writing – original draft, Visualization, Validation, Software, Methodology, Funding acquisition, Formal analysis, Data curation, Conceptualization. **Sandar Tin Tin:** Writing – review & editing, Supervision, Methodology, Funding acquisition, Data curation. **Edoardo Botteri:** Writing – review & editing, Supervision, Methodology, Conceptualization.

## Data availability

The datasets used in this study contain personal information and are not publicly available, but may be requested from the Breast Cancer Foundation New Zealand and Ministry of Health (NZ).

## Funding

Oliver Scott was supported by an 10.13039/501100001511Auckland Medical Research Foundation doctoral scholarship (Ref: 1217004).

This project was also supported by an 10.13039/501100001511Auckland Medical Research Foundation project grant (Ref: 1118017).

The funder had no role in the study design, data collection or analysis, decision to publish, or preparation of the manuscript.

## Declaration of competing interest

The authors declare that they have no known competing financial interests or personal relationships that could have appeared to influence the work reported in this paper.
