# Corrigendum to “Beta blocker use and breast cancer survival by subtypes: A population-based cohort study” [The Breast 81 (2025) 104474]

**DOI:** 10.1016/j.breast.2025.104478

**Published:** 2025-04-26

**Authors:** Oliver William Scott, Sandar Tin Tin, Edoardo Botteri

**Affiliations:** a Department of Oncology, School of Medical Sciences, University of Auckland, Auckland, New Zealand; b Department of Epidemiology and Biostatistics, School of Population Health, University of Auckland, Auckland, New Zealand; c Oxford Population Health, University of Oxford, Oxford, UK; d Department of Research, Cancer Registry of Norway, Norwegian Institute of Public Health, Oslo, Norway; e Section for Colorectal Cancer Screening, Cancer Registry of Norway, Norwegian Institute of Public Health, Oslo, Norway

The authors regret to inform readers that we have identified three instances of the phrasing “molecular subtype” used in the first version of the published manuscript (two in Fig. 1 and one in Supplementary Table 4). As microassays or other sophisticated methods of determining ER, PR, and HER2 statuses are not routinely carried out in New Zealand, the biomarkers used to determine breast cancer subtypes do not reflect tests carried out on a molecular level. As such, we have removed the word “molecular” and exclusively used “subtype” instead.

**Figure:**
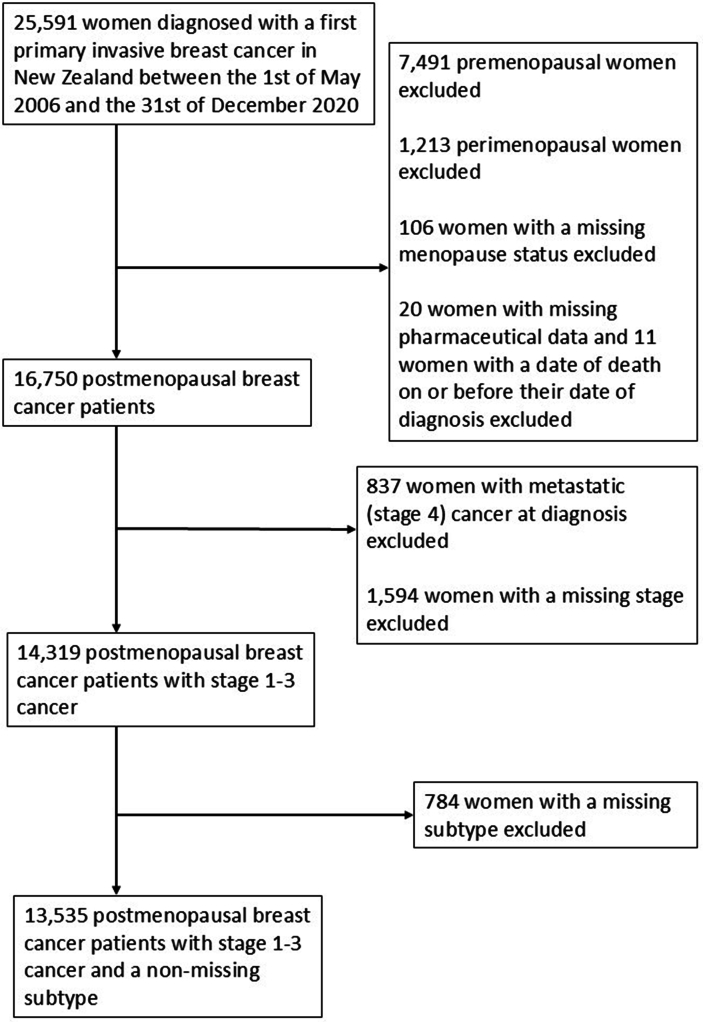

The authors would like to apologise for any inconvenience caused.

