# Supplementary material for: Corrigendum to “Beta blocker use and breast cancer survival by subtypes: A population-based cohort study” [The Breast 81 (2025) 104474]
Source: Breast. 2025 Apr 26;81:104478. doi: 10.1016/j.breast.2025.104478 (PMC12144928; doi:10.1016/j.breast.2025.104478)
Supplement: Multimedia component 1 [file mmc1.docx]

**Supplementary Table 1. Associations of breast cancer outcomes with peri-diagnostic use of beta blockers (vs non-use) in breast cancer patients, using a different exposure definition.**

|  | **Breast cancer specific death** | | | **Recurrence free interval** | | | **Distant recurrence free interval** | | |
| --- | --- | --- | --- | --- | --- | --- | --- | --- | --- |
| **Medication Usage At Diagnosis** | **No. Breast cancer deaths** | **No. person-years** | **Adjusted**^a^ **HR (95% CI)** | **No. Breast cancer deaths or recurrences** | **No. person-years** | **Adjusted**^a^ **HR (95% CI)** | **No. Breast cancer deaths or distant metastases** | **No. person-years** | **Adjusted**^a^ **HR (95% CI)** |
| **Overall association** |  |  |  |  |  |  |  |  |  |
| BB nonuser | 730 | 72,433 | 1.00 | 1,157 | 54,221 | 1.00 | 960 | 55,017 | 1.00 |
| BB user^b^ | 175 | 11,930 | 1.00 (0.83-1.20) | 241 | 9,190 | 0.92 (0.79-1.08) | 210 | 9,319 | 0.94 (0.79-1.11) |
| **By triple negative breast cancer** |  |  |  |  |  |  |  |  |  |
| BB nonuser, triple negative | 207 | 6,964 | 1.00 | 273 | 5,192 | 1.00 | 243 | 5,338 | 1.00 |
| BB user, triple negative^b^ | 48 | 1,254 | 0.84 (0.59-1.21) | 56 | 965 | 0.78 (0.56-1.08) | 51 | 985 | 0.76 (0.54-1.08) |

*^a^Adjusted for year of diagnosis, age, ethnic group, deprivation, urban/rural status, public/private status of the surgical treatment facility, register, size of the primary tumour, status of the regional lymph nodes, grade, mode of detection, lymphovascular invasion, subtype, and other drug use and hospitalised comorbidities (other drugs including statins, NSAIDs and aspirin, ACEIs, ARBs, and diuretics. Comorbidities including any cardiac condition as yes/no, diabetes, stroke, COPD, and peripheral vascular disease). Other drug covariates were modelled in the same fashion as beta blockers (i.e., requiring at least two dispensings in the six-month period prior to breast cancer diagnosis, including those dispensed on the date of diagnosis).*

*^b^In this analysis, women were required to have had at least two dispensings of a BB in the six-month period prior to diagnosis to be considered as a user. Women with only one dispensing in the six-month period prior to diagnosis were counted as nonusers.*

*Note) HR=Hazard Ratio, CI=Confidence Interval.*

**Supplementary Table 2. Associations of breast cancer outcomes with peri-diagnostic use of beta blockers (vs non-use) in breast cancer patients, using a different comparison group.**

|  | **Breast cancer specific death** | | | **Recurrence free interval** | | | **Distant recurrence free interval** | | |
| --- | --- | --- | --- | --- | --- | --- | --- | --- | --- |
| **Medication Usage At Diagnosis** | **No. Breast cancer deaths** | **No. person-years** | **Adjusted**^a^ **HR (95% CI)** | **No. Breast cancer deaths or recurrences** | **No. person-years** | **Adjusted**^a^ **HR (95% CI)** | **No. Breast cancer deaths or distant metastases** | **No. person-years** | **Adjusted**^a^ **HR (95% CI)** |
| **Overall association** |  |  |  |  |  |  |  |  |  |
| BB nonuser^b^ | 248 | 21,948 | 1.00 | 385 | 16,691 | 1.00 | 320 | 16,956 | 1.00 |
| BB user | 201 | 13,312 | 1.06 (0.87-1.30) | 274 | 10,225 | 0.97 (0.82-1.15) | 242 | 10,375 | 1.01 (0.84-1.21) |
| **By triple negative breast cancer** |  |  |  |  |  |  |  |  |  |
| BB nonuser, triple negative^b^ | 69 | 1,948 | 1.00 | 88 | 1,517 | 1.00 | 81 | 1,558 | 1.00 |
| BB user, triple negative | 52 | 1,441 | 0.84 (0.56-1.26) | 62 | 1,099 | 0.80 (0.56-1.15) | 57 | 1,127 | 0.76 (0.52-1.12) |

*^a^Adjusted for year of diagnosis, age, ethnic group, deprivation, urban/rural status, public/private status of the surgical treatment facility, register, size of the primary tumour, status of the regional lymph nodes, grade, mode of detection, lymphovascular invasion, subtype, and other drug use and hospitalised comorbidities (other drugs including statins, NSAIDs and aspirin, ACEIs, ARBs, and diuretics. Comorbidities including any cardiac condition as yes/no, diabetes, stroke, COPD, and peripheral vascular disease). Other drug covariates were modelled in the same fashion as beta blockers (i.e., yes/no in the four months prior to breast cancer diagnosis, including those dispensed on the date of diagnosis).*

*^b^In this analysis, BB users were compared to BB nonusers who used another antihypertensive medication (as opposed to comparing BB users to all BB nonusers). For this comparison, other antihypertensives included ‘Potassium Sparing Combination Diuretics’, ‘Thiazide and Related Diuretics’, ‘ACE Inhibitors’, ‘ACE Inhibitors with Diuretics’, ‘Angiotensin II Antagonists’, ‘Angiotensin II Antagonists with Diuretics’, ‘Dihydropyridine Calcium Channel Blockers’, ‘Other Calcium Channel Blockers’, ‘Alpha Adrenoceptor Blockers’, and ‘Centrally Acting Agents’.*

*Note) HR=Hazard Ratio, CI=Confidence Interval.*

**Supplementary Table 3. Associations of breast cancer outcomes with peri-diagnostic use of beta blockers (vs non-use) in breast cancer patients, using a different exposure definition and a different comparison group.**

|  | **Breast cancer specific death** | | | **Recurrence free interval** | | | **Distant recurrence free interval** | | |
| --- | --- | --- | --- | --- | --- | --- | --- | --- | --- |
| **Medication Usage At Diagnosis** | **No. Breast cancer deaths** | **No. person-years** | **Adjusted**^a^ **HR (95% CI)** | **No. Breast cancer deaths or recurrences** | **No. person-years** | **Adjusted**^a^ **HR (95% CI)** | **No. Breast cancer deaths or distant metastases** | **No. person-years** | **Adjusted**^a^ **HR (95% CI)** |
| **Overall association** |  |  |  |  |  |  |  |  |  |
| BB nonuser^b^ | 278 | 23,547 | 1.00 | 421 | 17,893 | 1.00 | 354 | 18,184 | 1.00 |
| BB user^c^ | 175 | 11,930 | 0.97 (0.79-1.19) | 241 | 9,190 | 0.91 (0.77-1.08) | 210 | 9,319 | 0.92 (0.76-1.10) |
| **By triple negative breast cancer** |  |  |  |  |  |  |  |  |  |
| BB nonuser, triple negative^b^ | 72 | 2,158 | 1.00 | 92 | 1,670 | 1.00 | 84 | 1,719 | 1.00 |
| BB user, triple negative^c^ | 48 | 1,254 | 0.89 (0.59-1.35) | 56 | 965 | 0.81 (0.56-1.17) | 51 | 985 | 0.79 (0.54-1.17) |

*^a^Adjusted for year of diagnosis, age, ethnic group, deprivation, urban/rural status, public/private status of the surgical treatment facility, register, size of the primary tumour, status of the regional lymph nodes, grade, mode of detection, lymphovascular invasion, subtype, and other drug use and hospitalised comorbidities (other drugs including statins, NSAIDs and aspirin, ACEIs, ARBs, and diuretics. Comorbidities including any cardiac condition as yes/no, diabetes, stroke, COPD, and peripheral vascular disease). Other drug covariates were modelled in the same fashion as beta blockers (i.e., requiring at least two dispensings in the six-month period prior to breast cancer diagnosis, including those dispensed on the date of diagnosis).*

*^b^In this analysis, BB users were compared to BB nonusers who used another antihypertensive medication (as opposed to comparing BB users to all BB nonusers). For this comparison, other antihypertensives included ‘Potassium Sparing Combination Diuretics’, ‘Thiazide and Related Diuretics’, ‘ACE Inhibitors’, ‘ACE Inhibitors with Diuretics’, ‘Angiotensin II Antagonists’, ‘Angiotensin II Antagonists with Diuretics’, ‘Dihydropyridine Calcium Channel Blockers’, ‘Other Calcium Channel Blockers’, ‘Alpha Adrenoceptor Blockers’, and ‘Centrally Acting Agents’.*

*^c^In this analysis, women were required to have had at least two dispensings of a BB in the six-month period prior to diagnosis to be considered as a user. Women with only one dispensing in the six-month period prior to diagnosis were counted as nonusers.*

*Note) HR=Hazard Ratio, CI=Confidence Interval.*

**Supplementary Table 4. Associations of breast cancer outcomes with peri-diagnostic use of beta blockers (vs non-use) in breast cancer patients, in pre and perimenopausal women only^a^.**

|  | **Breast cancer specific death** | | | **Recurrence free interval** | | | **Distant recurrence free interval** | | |
| --- | --- | --- | --- | --- | --- | --- | --- | --- | --- |
| **Medication Usage At Diagnosis** | **No. Breast cancer deaths** | **No. person-years** | **Adjusted^b^ HR (95% CI)** | **No. Breast cancer deaths or recurrences** | **No. person-years** | **Adjusted^b^ HR (95% CI)** | **No. Breast cancer deaths or distant metastases** | **No. person-years** | **Adjusted^b^ HR (95% CI)** |
| **Overall association** |  |  |  |  |  |  |  |  |  |
| BB nonuser | 641 | 52,996 | 1.00 | 1,045 | 38,313 | 1.00 | 860 | 39,192 | 1.00 |
| BB user | 21 | 1,626 | 1.03 (0.63-1.66) | 33 | 1,206 | 1.10 (0.75-1.61) | 32 | 1,210 | 1.24 (0.83-1.85) |
| **By triple negative breast cancer** |  |  |  |  |  |  |  |  |  |
| BB nonuser, triple negative | 143 | 5,608 | 1.00 | 178 | 4,056 | 1.00 | 160 | 4,191 | 1.00 |
| BB user, triple negative | 4 | 132 | 1.08 (0.38-3.12) | 5 | 101 | 0.91 (0.36-2.33) | 5 | 101 | 1.04 (0.41-2.68) |

*^a^This cohort consisted of 7,834 women (228 of which were users of BBs), and of these women, 826 had TNBC (19 of which were users of BBs).*

*^b^Adjusted for year of diagnosis, age, ethnic group, deprivation, urban/rural status, public/private status of the surgical treatment facility, register, size of the primary tumour, status of the regional lymph nodes, grade, mode of detection, lymphovascular invasion, subtype, and other drug use and hospitalised comorbidities (other drugs including statins, NSAIDs and aspirin, ACEIs, ARBs, and diuretics. Comorbidities including any cardiac condition as yes/no, diabetes, stroke, COPD, and peripheral vascular disease). Other drug covariates were modelled in the same fashion as beta blockers (i.e., yes/no in the four months prior to breast cancer diagnosis, including those dispensed on the date of diagnosis).*

*Note) HR=Hazard Ratio, CI=Confidence Interval.*
